# Social cognition as a mediator between neurocognition and functional outcome in early course schizophrenia

**DOI:** 10.1016/j.psychres.2025.116594

**Published:** 2025-06-15

**Authors:** Anju Kotwani, Jessica A. Wojtalik, Douglas D. Gunzler, Matthew J. Smith, Wilson J. Brown, Rochanne L. Honarvar, Martha Sajatovic, Matcheri S. Keshavan, Shaun M. Eack

**Affiliations:** a Jack, Joseph and Morton Mandel School of Applied Social Sciences, Case Western Reserve University, Cleveland, OH, USA; b Center for Healthcare Research and Policy, The MetroHealth System, Cleveland, OH, USA; c Population Health and Equity Research Institute, The MetroHealth System, Cleveland, OH, USA; d School of Medicine, Case Western Reserve University, Cleveland, OH, USA; e School of Social Work, University of Michigan, Ann Arbor, MI, USA; f Department of Psychological Sciences, Case Western Reserve University, Cleveland, OH, USA; g Neurological and Behavioral Outcomes Center, University Hospitals Cleveland Medical Center, Cleveland, OH, USA; h Department of Psychiatry, Beth Israel Deaconess Medical Center, Massachusetts Mental Health Center Division of Public Psychiatry, MA, USA; i Department of Psychiatry, Harvard Medical School, Boston, MA, USA; j School of Social Work, University of Pittsburgh, Pittsburgh, PA, USA; k Department of Psychiatry, University of Pittsburgh School of Medicine, Pittsburgh, PA, USA

**Keywords:** Schizophrenia, Early course, Neurocognition, Social cognition, Functional outcome, Mediation

## Abstract

Social cognition is an established mediator between neurocognition and functional outcome in schizophrenia, but there is limited evidence for this model specifically in the early course, which is a critical window for intervention to reduce long-term disability. This study aimed to test the social cognition mediator model in the early course of schizophrenia and identify social cognitive subdomains that may have stronger indirect effects on the relationship between neurocognition and functional outcome. This secondary analysis utilized baseline cognitive and functional outcome data from a cognitive remediation trial for outpatients with early course schizophrenia (*N* = 102). A path analysis approach was used to compute mediation of social cognition (mediator; composite index and subdomain scores) on the relationship between neurocognition (predictor; composite index) and functional outcome (outcome; composite index). Significant positive associations were observed between neurocognition, social cognition, and functional outcome. As previously observed, the mediation effect of the social cognition composite was significant (*p* = 0.042). Of the seven social cognitive subdomains, only social inference (*p* < 0.001) emerged as a significant mediator. Reverse mediation models (mediator; neurocognition) were non-significant. Results suggest that the impact of cognition on functional outcome in the early course occurs, in part, through the impact of neurocognition on social cognition, which subsequently influences functional outcome. In addition, social inference is possibly an important treatment target of functional recovery in the early course of the condition, highlighting a potential future research direction. These findings further support the provision of comprehensive cognitive remediation approaches to facilitate functional recovery.

## 1. Introduction

Nearly 30 years of research have established that both neuro- and social cognitive challenges are significant barriers to functional recovery from schizophrenia (Green, 1996; Green et al., 2019). As such, comprehensive cognitive remediation interventions that address both cognitive domains are highly promising for improving functional outcome, particularly in the early course of the condition (Revell et al., 2015; Wojtalik et al., 2022). Empirical evidence from studies in more chronic phases of schizophrenia demonstrate an interaction among the primary targets of comprehensive cognitive remediation such that social cognition significantly mediates the relationship between neurocognition and functional outcome (Halverson et al., 2019). However, this mediational model has yet to be replicated in early course schizophrenia, and such research is critical for continued optimization of cognitive remediation specific to this phase of the condition to reduce the likelihood of long-term disability.

The degree of neurocognitive impairment among individuals with schizophrenia is estimated to be at least two standard deviations below that of healthy individuals (Heinrichs and Zakzanis, 1998; Keefe et al., 2011) and a standard deviation below for social cognition (Savla et al., 2013). In the early course of schizophrneia, neurocognitive and social cognitive impairments are present, of substantial magnitude, and significantly contribute to poor functional outcome (Healey et al., 2016; Mesholam-Gately et al., 2009). The relationship between neuro- and social cognition and their subsequent influence on functional outcome in schizophrenia has garnered attention since the late 1990′s (Green and Nuechterlein, 1999; Kee et al., 1998). This body of research concludes that, while neurocognition and social cognition share significant variance, they are distinct and separable constructs (Allen et al., 2007; van Hooren et al., 2008). Fett et al. (2011) conducted the original meta-analysis on the relationship between neurocognition, social cognition, and functional outcome in schizophrenia. Across 52 studies (2692 patients), their results demonstrated that, in general, community functioning has a more pronounced association with social cognition compared to neurocognition (Fett et al., 2011). A more recent meta-analysis in the early course population (46 studies; 3767 patients) further affirms that social cognition exhibits a stronger association with functional outcome, both cross-sectionally and longitudinally, relative to neurocognitive subdomains (Cowman et al., 2021).

Considering the significance of both neurocognition and social cognition as predictors of functional outcome in schizophrenia, albeit a stronger association for social cognition (Fett et al., 2011), researchers subsequently explored the mechanistic interplay between these variables. Schmidt et al. (2011) conducted one of the first empirical reviews (*N* = 1308) and primary studies (*N* = 148, average illness duration = 10.3 years) of social cognition as a mediator between neurocognition and functional outcome in schizophrenia. In 14 of the 15 included studies, which included their own study of 148 patients, social cognition was a significant mediator with an average indirect effect size of 0.20 (range: 0.11–0.28, standardized indirect effect size calculated from nine of the included studies that provided adequate information). This review also demonstrated that subdomains of social cognition differentially mediate the impact of neurocognition on functional outcome. Social knowledge (0.28; awareness of norms) presented the largest average indirect effect size, followed by social perception (0.21; reading social cues), emotion perception (0.19; identify and respond to emotions in others), and theory of mind (ToM; 0.14; understanding others’ mental states). More recently, Halverson et al. (2019) conducted a meta-analysis of 166 studies across 12,868 patients with schizophrenia with an average illness duration of 16 years (SD = 7.64) that provided continued evidence of the significant role of social cognition as a mediator between neurocognition and functional outcome (*c*′=0.14, *p* < 0.01).

Overall, there is a mechanistic pathway to poor functional outcome in schizophrenia, influenced at least in part by the impact of impaired neurocognition on social cognition. What remains less clear is the degree to which this mediational relationship is a pathway to poor functional outcome in the early course of schizophrenia. For instance, in a sample of individuals at high risk for psychosis, the indirect effect of social cognition between neurocognition and functional outcome was not significant (Barbato et al., 2013). While the social cognition mediator model is well-studied in chronic schizophrenia, its applicability to the early course phase remains uncertain, especially given the lack of a significant model in the high-risk population. The current study aimed to test the social cognition mediator model in early course schizophrenia and identify any social cognitive subdomains that have a particularly strong indirect influence on functional outcome.

## 2. Methods

### 2.1. Procedure

Baseline data used for the current research was derived from a two-site (Boston, MA [*n* = 49]; Pittsburgh, PA [*n* = 53]) 18-month randomized confirmatory trial of Cognitive Enhancement Therapy (CET) for outpatients with early-course schizophrenia (*N* = 102), described in more detail in Wojtalik et al. (2022). The parent trial was conducted from June 2012 to May 2018, was approved annually by the Beth Israel Deaconess Medical Center (Boston, MA) and University of Pittsburgh (Pittsburgh, PA) institutional review boards, and all participants provided written informed consent. Participants were recruited from well-established, community-based early course psychosis treatment programs serving the Boston and Pittsburgh areas. These university-affiliated outpatient clinics provide a range of standard outpatient mental health services for the early course population, such as medication management, case management, individual counseling, support groups, and peer support services. The research team collaborated closely with clinic providers and staff to facilitate recruitment. Participants continued their usual care services during study participation, and the research clinicians maintained communication with the participants’ regular providers as needed. Included participants completed a comprehensive battery of cognitive and functional outcome measures at baseline, 9- and 18-month time points. The baseline (i.e., pre-treatment) data was used for this research to test the social cognition mediator model in early course schizophrenia.

### 2.2. Participants

The eligibility criteria for the parent trial included: a) a schizophrenia diagnosis (as well as schizoaffective or schizophreniform disorders) confirmed by the Structured Clinical Interview for the DSM-IVTR (First, 2002), b) onset of psychotic symptoms within the past 10 years, c) age range of 18–55, d) IQ ≥ 80, e) clinically stable positive symptoms, f) adherence to prescribed antipsychotic medication, and g) ability to read and speak fluent English. It is noteworthy that the 10-year illness duration criterion is consistent with the range of definitions used in the literature for early course schizophrenia (Newton et al., 2018). The full baseline sample of 102 participants included in the parent trial (Wojtalik et al., 2022) was used for this research, and the baseline demographic and clinical factors of the sample are presented in Table 1.

### 2.3. Measures

All cognitive and functional outcome measures below are standard and reliable measures commonly used in the schizophrenia literature.

#### 2.3.1. Neurocognition composite (Predictor)

Neurocognition was measured with the NIMH MATRICS Consensus Cognitive Battery (MCCB; Nuechterlein et al., 2008). This standardized, reliable, and validated tool includes paper-and-pencil and computer-administered tests to assess the cognitive subdomains most impaired by the condition of schizophrenia, including processing speed, attention/vigilance, working memory, verbal learning, visual learning, problem solving, and social cognition. A neurocognitive composite score was calculated from the subdomains listed above, excluding social cognition, to *T*-scores with a *M* = 50 (*SD* = 10) adjusted for sex and age (Kern et al., 2008). A higher neurocognitive composite *T*-score indicates better neurocognitive performance.

#### 2.3.2. Social cognition composite and subdomains (Mediator)

##### Emotional Intelligence.

The Mayer-Salovey-Caruso Emotional Intelligence Test (MSCEIT; Mayer et al., 2003) is a 141-item standardized computer-administered and performance-based assessment of emotion processing and management that yields four subscale scores: perceiving, facilitating, understanding, and managing emotions. Sub-scale scores are based on a large normative sample scale (Mayer et al., 2003: M = 100, *SD* = 15), and a higher score represents stronger emotional intelligence. The MSCEIT is recommended by the NIMH MATRICS committee for assessing social cognition in schizophrenia (Green et al., 2006) because of its strong reliability and validity in this population (Eack et al., 2010; Mayer et al., 2003; Nuechterlein et al., 2008; Wojtalik et al., 2013).

##### Emotion Recognition.

The Penn Emotion Recognition Test-40 (ER-40; Kohler et al., 2003) measures the ability to accurately identify different emotions (i.e., happy, sad, anger, and fear) across 40 colored images of human faces with a computer-administered, performance-based, and forced choice format (Kohler et al., 2003). This test generates a total score of correctly identified emotions (Kohler et al., 2003) and is a frequently used measure in schizophrenia with good reliability and validity (Carter et al., 2009).

##### Social Inference.

The Awareness of Social Inference Test (TASIT; McDonald et al., 2003) is a performance-based assessment that includes 16 short (15 to 60 s) vignettes portraying social exchanges between actors. After watching a vignette, participants are asked four questions regarding their perceptions of lies or sarcasm in the social exchange. A total score is generated from correct responses, with higher scores indicative of stronger social inference ability (McDonald et al., 2003).

The TASIT is a frequently used measure in clinical populations with good reliability and construct validity (McDonald et al., 2006).

##### Theory of Mind (ToM).

The Hinting Task is a performance-based measure for people with schizophrenia (Corcoran et al., 1995). Ten short stories are narrated to participants, and the total score of correct responses reflects the ability to correctly perceive the hint of the character’s true intention. The hinting task is a frequently used measure in schizophrenia with adequate test-retest reliability and internal consistency (Klein et al., 2020).

##### Social Cognition Composite.

An overall social cognitive composite index was computed from seven individual scores from the aforementioned measures (four MSCEIT subscale scores and total scores from the ER-40, TASIT, and Hinting Task). The individual measure scores were converted to a common Z metric, averaged, and scaled to a *T*-score with a *M* of 50 (*SD* = 10) such that higher scores indicate better overall social cognitive performance.

#### 2.3.3. Functional outcome composite (Outcome)

Functional outcome assessments included the Major Role Adjustment Inventory (MRAI; Hogarty et al., 1974), Social Adjustment Scale-II (SAS-II; Schooler et al., 1979), Global Assessment of Functioning (GAF; Endicott et al., 1976), and the social contacts (item 2) and usefully employed (item 3) items from the Strauss-Carpenter Outcome Scale (SCOS; Strauss and Carpenter Jr., 1972). These measures cover the functional subdomains of global functioning, social functioning, independent living, and employment (see Wojtalik et al., 2022).

The neurocognitive (Cronbach’s *α* = 0.74) and social cognitive (Cronbach’s *α* = 0.73) composites demonstrated adequate reliability within the current sample, and the functional outcome composite exhibited good reliability (Cronbach’s *α* = 0.81).

### 2.4. Data analysis

The mediation effect of social cognition (mediator) on the relationship between neurocognition (predictor) and functional outcome (outcome) in early course schizophrenia was tested using a model-based, path analysis approach in RStudio (version 4.2.0) with the ‘mediation’ package (Tingley et al., 2014). All analyses adjusted for study location (Pittsburgh, PA vs. Boston, MA). Unlike the path analysis approach outlined by Baron and Kenny (Baron and Kenny, 1986), which requires a significant relationship between the predictor and outcome variable (path c) before proceeding with mediation model building, modern approaches to mediation indicate that a significant total effect between the predictor and the outcome variables is not required, especially if there is a strong theoretical support for their relationship (Rucker et al., 2011; Shrout and Bolger, 2002). As presented in the introduction, there is strong evidence for the social cognition mediator model in schizophrenia (Halverson et al., 2019), where neurocognition influences social cognition, which subsequently impacts functional outcome. Following the steps outlined by Tingley et al. (2014), the first step in this mediation analysis was fitting a linear model of the relationship between the mediator and predictor variables (i.e., path a: social cognition ~ neurocognition), referred to as the *mediator model* (Tingley et al., 2014). A total of eight mediator models were executed to test for significant associations between the neurocognitive composite and the social cognitive composite and the seven social cognitive subdomain scores (i.e., four emotional intelligence subscales, emotion recognition, social inference, and ToM). Model building did not proceed for social cognitive variables not significantly associated with neurocognition, consistent with the assumptions of mediation analysis (Shrout and Bolger, 2002), including modern approaches (Tingley et al., 2014).

The second step in this approach is fitting a linear model with the outcome variable as a function of the predictor and mediator variables (i.e., paths b and c′: functional outcome ~ neurocognition + social cognition), referred to as the *outcome model* (Tingely et al., 2014). This second set of models tests the relationship between social cognition scores (i.e., those significant in step 1) and the functional outcome composite while adjusting for the neurocognitive composite. A significant effect of social cognition in the outcome model indicates possible mediation and thus permits the final step to test for significance of mediation. The final step uses the ‘mediate’ function to combine the fitted mediator and outcome models to calculate the mediation effect of social cognition on the relationship between neurocognition and functional outcome. The mediation effect is quantified as the product of the estimated coefficients of social cognition from the mediator and outcome models (i.e., path a * path b). The direct effect (path c′), which is the effect of neurocognition on functional outcome, while controlling for social cognition, is also provided. As recommended by Preacher & Hayes (2004), bootstrapping was conducted with 5000 permutations using the bias-corrected and accelerated option for confidence interval calculation to test for significance of the mediation effect and direct effect. Finally, reverse mediation effects (Fiedler et al., 2011) were examined to rule out the possibility of neurocognition as a mediator of the relationship between social cognition and functional outcome following the same steps outlined above.

## 3. Results

Table 2 presents the results for the association between neurocognition (predictor) and social cognition (mediator) in early course schizophrenia (path a) within each of the eight mediation models. The social cognitive and neurocognitive composites were observed to have a significant positive relationship (Model 1), such that higher neurocognitive composite scores were predictive of higher social cognitive scores. Regarding the specific subdomains of social cognition, significant positive associations were demonstrated between the neurocognition composite and the understanding (Model 4) and managing (Model 5) emotions MSCEIT subscales, as well as emotion recognition (Model 6), social inference (Model 7), and ToM (Model 8), with higher neurocognitive composite scores predicting better social cognitive subdomain performance. The MSCEIT subscales of perceiving (Model 2) and using (Model 3) emotions were not significantly associated with the neurocognitive composite (Table 2), and thus the next step of model building did not proceed for these subscales. After adjusting for multiple comparisons using a Bonferroni correction, the five significant uncorrected models above (Models 1 and 4 through 8) remained significant (Model 1: *p* < 0.001; Model 4: *p* < 0.001; Model 5: *p* < 0.001; Model 6: *p* = 0.015; Model 7: *p* < 0.001), except for Model 8 (*p* = 0.058), which was slightly above the conventional significance level. Given the exploratory nature of exploring social cognitive subdomains as mediators, the ToM model (Model 8) was carried forward to the second step of model building.

**Fig. 1.**
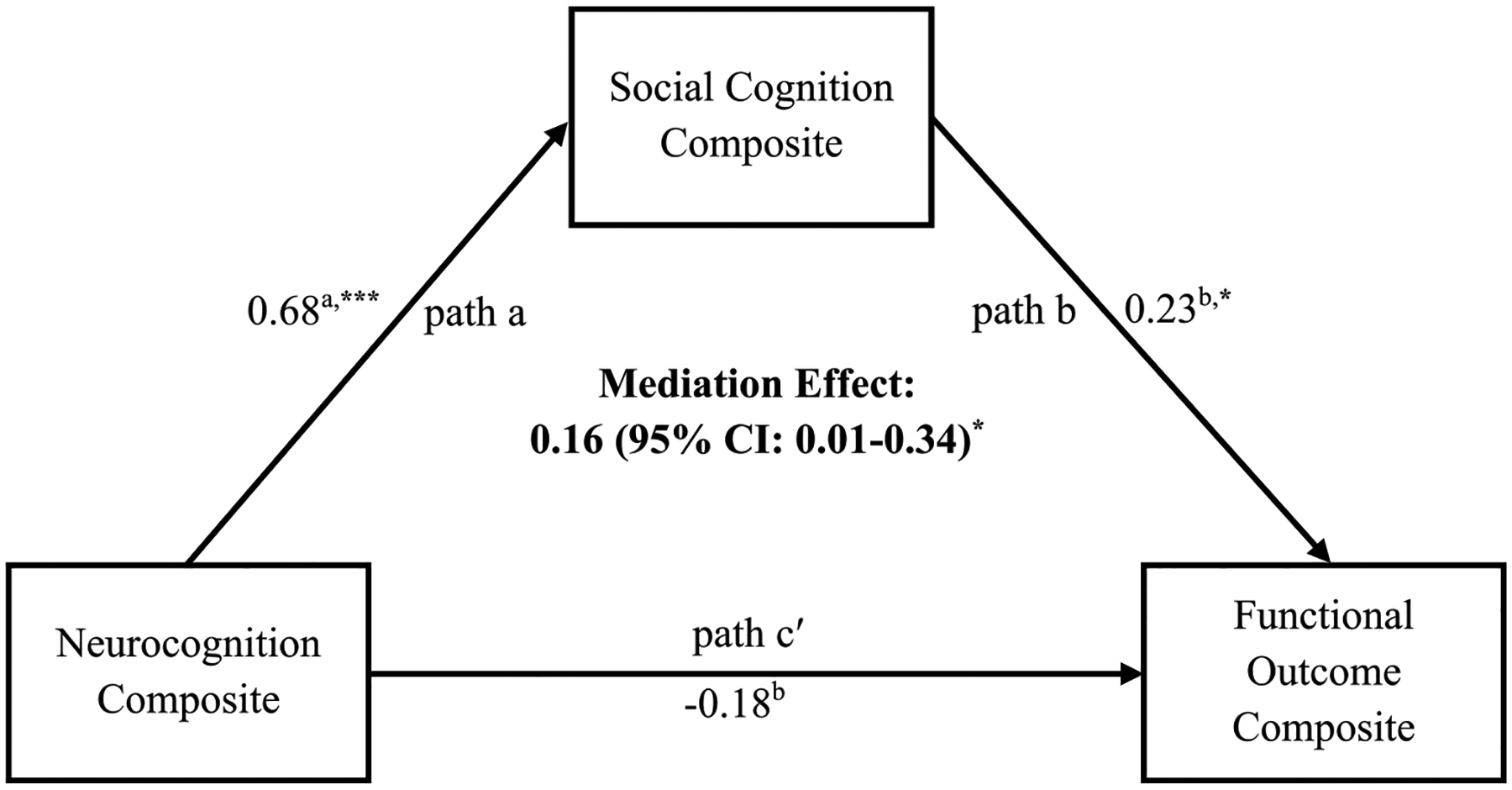
Overall Social Cognition as a Significant Mediator between Neurocognition and Functional Outcome (*Note*. Mediation Effect [0.68×0.23 = 0.16], * = *p* < 0.05, *** = *p* < 0.001, ^a^mediator model [see Table 2], ^b^outcome model [see Table 3]).

**Fig. 2.**
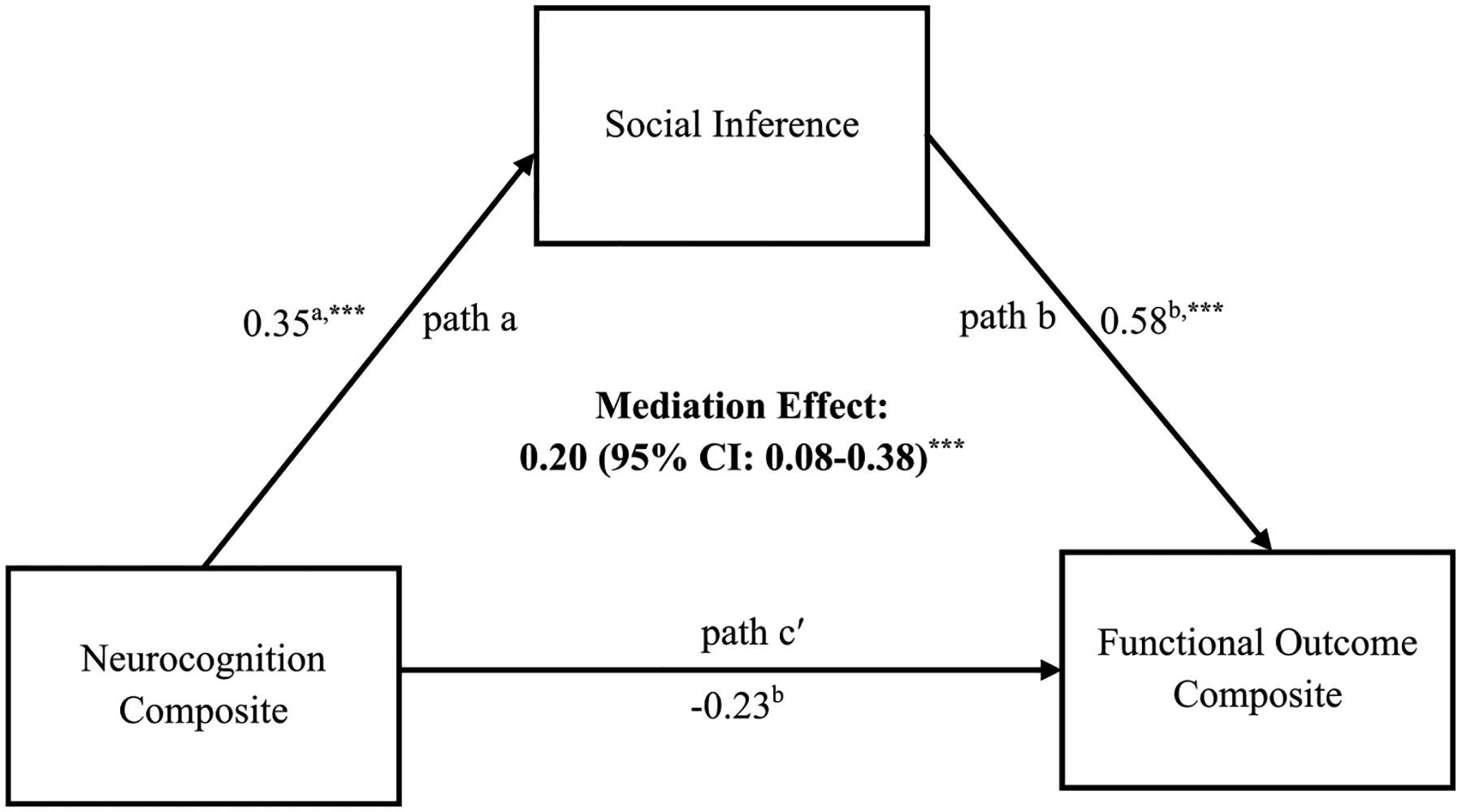
Social Inference as a Significant Mediator between Neurocognition and Functional Outcome (*Note*. Mediation Effect, [0.35×0.58 = 0.20], ***=*p* < 0.001, ^a^mediator model [see Table 2], ^b^outcome model [see Table 3]).

Table 3 displays the results of the six fitted outcome models. The social cognition composite (Model 1) and the social inference subdomain (Model 5) demonstrated significant and positive associations with functional outcome, while adjusting for neurocognition (i.e., path b). As observed in Fig. 1 for the social cognition composite, the mediation effect was significant (*p* = 0.042), while the direct effect was non-significant (path c′: −0.18 [95 % CI: 0.44, 0.14], *p* = 0.221). Similarly for social inference (see Fig. 2), the mediation effect was significant (*p* < 0.001) and slightly larger relative to the mediator effect of the social cognitive composite, while the direct effect was not significant (path c′: 0.23 [95 % CI: 0.50, 0.01], *p* = 0.076). Such results indicate that the relationship between overall neurocognitive functioning on functional outcome is not direct, but rather significantly mediated by overall social cognitive ability, particularly social inference, in early course schizophrenia. No other subdomains of social cognition demonstrated an effect on functional outcome (Table 3) and thus did not meet the conditions to test for significance of mediation.

Finally, sensitivity analyses tested reverse mediation effects of neurocognition (mediator) on the relationship between social cognition (predictor) and functional outcome (outcome). The relationship between neurocognition on functional outcome while adjusting for social cognition was non-significant (path b in this reverse model), violating the assumption of mediation model building. However, to further confirm the absence of reverse mediation effects for neurocognition, the mediating effect of neurocognition on the relationship between social cognition and functional outcome was calculated and observed to be non-significant for the social cognition composite (‒0.06 [95 % CI: −0.16, 0.05], *p* = 0.222) and social inference subdomain (− 0.10 [95 % CI: ‒0.23, 0.01], *p* = 0.084). The direct effect (path c′ in reverse model) for the social cognition composite and social inference subdomain were significant, as they are replicated results from the original outcome model (i.e., path b, see Table 3). These reverse mediation results support the social cognitive mediator findings (Figs. 1 and 2) where neurocognition precedes social cognition, which subsequently impacts functional outcome.

## 4. Discussion

Several studies over the last decade have provided strong evidence for the mediating role of social cognition in the relationship between neurocognition and functional outcome in chronic schizophrenia (Halverson et al., 2019). However, there is a limited understanding of how neurocognition and social cognition interact to influence functional outcome in the early phases of schizophrenia. Therefore, the current research aimed to test whether the social cognition mediator model holds in early course schizophrenia and identify any subdomains of social cognition that may have stronger indirect effects. Understanding the social cognition mediator model in early course schizophrenia is crucial for continued enhancement of early intervention programs, particularly cognitive remediation.

This research provides evidence that a significant mediation effect of overall social cognition between neurocognition and functional outcome is present in early course schizophrenia, consistent with previous meta-analytic evidence in chronic schizophrenia (Halverson et al., 2019). In a study of first-episode psychosis, non-significant relationships were observed at baseline between cognition and functional outcome, but significant relationships emerged at a two-year follow-up, with social cognition significantly mediating the relationship between verbal memory and functional outcome (Gonzalez-Ortegá et al., 2020). Given the trajectory of impairment in schizophrenia (García-Fernández et al., 2020), it is possible that the mediational role of social cognition on functional outcome becomes more pronounced as illness progresses, especially given that the current study focused on the early course rather than first-episode patients. Future research is needed to elucidate the progression of this social cognitive mediator model across different phases of illness to identify the most critical time frame for cognitive intervention.

The significant social cognition mediator model in the current study supports comprehensive cognitive remediation approaches to facilitate functional recovery from schizophrenia (Eack, 2012; Green et al., 2019; Peña et al., 2016; Sampedro et al., 2021). Meta-analytic evidence demonstrates that short-term neurocognitive training alone has minimal impact on functional outcome (i.e., Hedge’s *g* = 0.21; Lejeune et al., 2021; McGurk et al., 2007). In contrast, programs such as Cognitive Enhancement Therapy (CET; Hogarty et al., 2004), which integrates 18 months of computer-based neurocognitive training and social cognitive groups, have found significant medium-to-large effects on cognition and functional outcome in early course schizophrenia (Cohen’s *d*, 0.35–1.55; Eack et al., 2009; Wojtalik et al., 2022). Overall, this evidence underscores the need for further research into the mechanistic relationships among cognitive domains and functional outcome in the early course population. Such research is vital for optimization of effective, early course treatment programs, such as coordinated specialty care (Dixon et al., 2018; Lewandowski, 2021). Early intervention with comprehensive cognitive remediation programs that target functional recovery have the potential to alter the chronic trajectory of schizophrenia, leading to better long-term outcome (Srihari and Keshavan, 2022).

Results also revealed that the social inference subdomain distinctly influences the relationship between neurocognition and functional outcome in early course schizophrenia. Social inference is the ability to understand and utilize information in the social environment, such as verbal and non-verbal social cues and knowledge of roles and norms, in order to respond appropriately across different social interactions. People living with schizophrenia, including those in the early course (Addington et al., 2006), experience considerable challenges with social inference ability (Green et al., 2019; Healey et al., 2016; Savla et al., 2013) that negatively impact functioning (Couture et al., 2006; Horan et al., 2012). For instance, people with schizophrenia may struggle with understanding social norms in group contexts, such as work meetings or school projects. They may not recognize when it is time to remain quiet for others to speak or will not contribute when asked, all of which can result in anxiety, disengagement, or exclusion. Programs, such as CET, target social inference through several sessions where participants learn and practice how to engage in appropriate behaviors based on varying social contexts. For example, CET includes a session on social context appraisal where participants learn how to look for verbal and nonverbal clues in a social situation (e.g., job interview vs. spending time with a long-time friend) to inform if they should be using formal (’front stage’) or informal (’backstage’) behavior (Hogarty and Greenwald, 2006). Another example of a social inference exercise used in Social Cognitive Enhancement Training is an activity where participants use social cue information to correctly sequence cartoon panels of a social situation (Choi and Kwon, 2006). Despite the strong association with real-life functioning, social inference is understudied in the early course population relative to other social cognitive subdomains (e.g., ToM; Healey et al., 2016). Social inference may be a key treatment target for early course schizophrenia, and further research is needed to better understand its relative contribution to functional outcome.

It is noteworthy that ToM, emotion recognition, and emotional intelligence did not have a significant indirect effect on the relationship between neurocognition and functional outcome. This is somewhat surprising given the well-established associations between these subdomains of social cognition and functional outcome in schizophrenia, including the early course population (Horan et al., 2012; Pinkham et al., 2007). It may be that social cognitive impairments in these subdomains, with the exception of social inference, are less pronounced in the early stages of the illness compared to chronic schizophrenia (García-Fernández et al., 2020; Savla et al., 2013). Although, the study by Gonzalez-Ortegá et al. (2020) observed a significant mediation effect of social cognition at the two-year follow-up in first-episode psychosis using the MSCEIT to assess social cognition—the same measure of emotional intelligence used in the current study. Thus, differences in neurocognitive and functional outcome assessments may account for variations in the results observed between the present study and prior research (i.e., Gonzalez-Ortegá et al. 2020).

This study requires interpretation within the context of inherent limitations. First, the present analysis was cross-sectional; yet replication of this model in early course schizophrenia was primary aim of the current study. Additionally, this analysis utilized baseline data from a parent trial designed to confirm effectiveness of CET on specific domains of cognition and functional outcome (Wojtalik et al., 2022). The secondary nature of this analysis may introduce bias as data were not primarily collected with intent to examine this social cognitive mediator model. Further, the current sample may not fully represent individuals living with early course schizophrenia given the inclusion criteria used for the parent trial, such as medication adherence, stability of clinical symptoms, and an IQ ≥ 80. Moreover, the use of a broader 10-year illness duration criterion, while adherent to the technical definition of early course schizophrenia (Newton et al., 2018), may dilute obtained results and contribute to non-significant mediation effects. However, this may be less of a concern given the majority (76 %) of the sample had an illness duration under five years. Given that psychosis often goes untreated for more than a year following initial onset in the community (Addington et al., 2015), the parent study adopted a broader definition of early course schizophrenia to include individuals in a recovery phase who were more likely to engage in a long-term intervention (Wojtalik et al., 2023). This broader definition may enhance the external validity of the current findings by increasing applicability to a more representative and diverse clinical population. However, considering the non-significant social cognition mediation effect in the clinical high-risk population (Barbato et al., 2013) and non-significant relationships between cognition and functional outcome in first episode psychosis (Gonzalez-Ortegá et al., 2020), it is possible that the indirect effect of social cognition on functional outcome strengthens with increased illness duration. Future research would benefit from clarifying the longitudinal trajectory of the social cognition mediator model by examining it across narrower definitions of early course schizophrenia. Such research would guide the personalization of cognitive interventions to illness phase and support early preventive strategies to preserve social cognitive functioning from the earliest phases of the condition. Additionally, this study did not include all the subdomains of social cognition including attribution bias or empathy. Lastly, while testing the mediation effects of social cognitive subdomains is a strength of this research, composite indexes for neurocognition and functional outcome were utilized to preserve statistical power, given the limited research of this social cognitive mediator model in the early course population. Future research should employ more complex analyses (e.g., structural equation modeling) to examine specific subdomains of neurocognition, social cognition, and functional outcome. This approach will provide a more nuanced understanding of these relationships and better inform the development of more targeted treatment strategies for early course schizophrenia. Future studies may benefit from including the full range of individual subdomains of social cognition including attribution bias and empathy and using an alternative model to explain mediation effect of social cognition on functioning through social competence (i.e., applied social skills; Couture et al., 2011).

In conclusion, the findings of this study provide support for the social cognition mediator model in early course schizophrenia, as established in more chronic populations (Halverson et al., 2019), and identify social inference as a potentially influential subdomain of social cognition on functional outcome. These findings both support the need for comprehensive cognitive remediation approaches and highlight the potential for future research to explore more complex, longitudinal social cognitive mediator models within the early course. Such research will further clarify the mechanistic relationships among subdomains of cognition and functional outcome, ultimately guiding the development and adaptation of early course psychosis interventions that target functional recovery.

## Figures and Tables

**Table 1:** Baseline sample characteristics of outpatients with early course schizophrenia (*N* = 102).

Variable	*M* (*SD*)/*N* (%)
Age	24.76 (5.44)
Sex (% Male)	76 (75 %)
Race^a^ (% Non-white)	44 (43 %)
IQ Score	107.83 (10.40)
Education^b^ (% Some college)	69 (73 %)
Employed^c^ (% Not employed)	70 (69 %)
Illness length^c^ (years)	3.69 (2.28)
Schizophrenia diagnosis (Number with)	82 (80 %)
Past substance use diagnosis (Number with)	50 (49 %)
Antipsychotic Medication Dose^d^	429.60 (331.70)
Antipsychotic Medication Adherence^c^ (Number adherent)	75 (74 %)
Study Location: Boston	49 (48 %)
Pittsburgh	53 (52 %)
Neurocognition Composite	40.78 (7.26)
Functional Outcome Composite	50.03 (9.95)
Social Cognition Composite	50.14 (10.06)
MSCEIT Perceiving Emotions^c,e^	99.81 (19.87)
MSCEIT Using Emotions^e,f^	95.25 (15.74)
MSCEIT Understanding Emotions^c,e^	90.18 (12.24)
MSCEIT Managing Emotions^c,e^	90.16 (12.49)
Emotion Recognition^g,h^	32.69 (3.45)
Social Inference^c,i^	50.95 (6.68)
Theory of Mind^c,j^	14.13 (3.28)

*Note*. CPZ = Chlorpromazine; IQ = Intelligence Quotient; MSCEIT = Mayer-Salovey-Caruso Emotional Intelligence Test (Mayer et al., 2003).

a Of those who identified their race as non-white, 24 (56 %) indicated their race as African American, seven (16 %) as more than one race, six (14 %) as Other, five (12 %) as Asian, one (2 %) as Hawaiian/Pacific Islander, and one (2 %) as Hispanic.

b Total sample of 94 with available data.

c Total sample of 101 with available data.

d Total sample of 98 with available data.

e Scored scaled to a normative sample with a *M* of 50 (*SD* = 10).

f Total sample of 100 with available data.

g Total sample of 95 with available data.

h Penn Emotion Recognition Task, correct responses (Kohler et al., 2003).

i The Awareness and Social Inference Test (TASIT), total score (McDonald et al., 2003).

j Hinting Task, total score (Corcoran et al., 1995).

**Table 2:** Mediator models (Step 1): examining the associations between neurocognition and Social Cognition (path a).

Social Cognition ~ Neurocognition	*b*	*SE*	*t*	*p*
Model 1: Social Cognition Composite	0.68	0.12	5.63	<0.001
Model 2: MSCEIT Perceiving Emotions	0.45	0.27	1.67	0.098
Model 3: MSCEIT Using Emotions	0.25	0.23	1.10	0.272
Model 4: MSCEIT Understanding Emotions	0.76	0.15	5.04	<0.001
Model 5: MSCEIT Managing Emotions	0.81	0.15	5.31	<0.001
Model 6: Emotion Recognition	0.15	0.05	3.21	0.002
Model 7: Social Inference	0.35	0.08	4.10	<0.001
Model 8: Theory of Mind	0.12	0.04	2.74	0.007

*Note*. All models adjusted for study location. MSCEIT = Mayer-Salovey-Caruso Emotional Intelligence Test (Mayer et al., 2003).

**Table 3:** Outcome Models (Step 2): Examining Associations between Social Cognition (Mediator) and Functional Outcome (Outcome) while Adjusting for Neurocognition (Predictor) (paths b & c′).

Functional Outcome ~ Neurocognition + Social Cognition	*b*	*SE*	*t*	*p*
Model 1:				
Neurocognition Composite	−0.18	0.15	−1.23	0.222
**Social Cognition Composite**	**0.23**	**0.11**	**2.18**	**0.032**
Model 2:				
Neurocognition Composite	−0.10	0.15	−0.67	0.505
MSCEIT Understanding Emotions	0.10	0.09	1.12	0.265
Model 3:				
Neurocognition Composite	−0.11	0.15	−0.77	0.445
MSCEIT Managing Emotions	0.11	0.09	1.28	0.203
Model 4:				
Neurocognition Composite	−0.07	0.14	−0.48	0.633
Emotion Recognition	0.16	0.31	0.53	0.601
Model 5:				
Neurocognition Composite	−0.23	0.13	−1.71	0.090
**Social Inference**	**0.58**	**0.14**	**4.01**	**<0.001**
Model 6				
Neurocognition Composite	−0.06	0.14	−0.48	0.634
Theory of Mind	0.35	0.31	1.14	0.258

*Note*. All models adjusted for study location. MSCEIT = Mayer-Salovey-Caruso Emotional Intelligence Test (Mayer et al., 2003).
